# Protealysin Targets the Bacterial Housekeeping Proteins FtsZ and RecA

**DOI:** 10.3390/ijms231810787

**Published:** 2022-09-15

**Authors:** Olga Tsaplina, Sofia Khaitlina, Ksenia Chukhontseva, Maria Karaseva, Ilya Demidyuk, Irina Bakhlanova, Dmitry Baitin, Tatiana Artamonova, Alexey Vedyaykin, Mikhail Khodorkovskii, Innokentii Vishnyakov

**Affiliations:** 1 Institute of Cytology, Russian Academy of Sciences, 194064 St. Petersburg, Russia; 2 Institute of Molecular Genetics of National Research Centre “Kurchatov Institute”, 123182 Moscow, Russia; 3 Kurchatov Genome Center—PNPI, Petersburg Nuclear Physics Institute Named by B.P. Konstantinov of National Research Centre ‘‘Kurchatov Institute”, 188300 Gatchina, Russia; 4 Department of Nanobiotechnologies, Peter the Great St. Petersburg Polytechnic University, 195251 St. Petersburg, Russia

**Keywords:** protealysin, RecA, FtsZ, *Serratia proteamaculans*, interbacterial competition

## Abstract

*Serratia proteamaculans* synthesizes the intracellular metalloprotease protealysin. This work was aimed at searching for bacterial substrates of protealysin among the proteins responsible for replication and cell division. We have shown that protealysin unlimitedly cleaves the SOS response protein RecA. Even 20% of the cleaved RecA in solution appears to be incorporated into the polymer of uncleaved monomers, preventing further polymerization and inhibiting RecA ATPase activity. Transformation of *Escherichia coli* with a plasmid carrying the protealysin gene reduces the bacterial UV survival up to 10 times. In addition, the protealysin substrate is the FtsZ division protein, found in both *E. coli* and *Acholeplasma laidlawii*, which is only 51% identical to *E. coli* FtsZ. Protealysin cleaves FtsZ at the linker between the globular filament-forming domain and the C-terminal peptide that binds proteins on the bacterial membrane. Thus, cleavage of the C-terminal segment by protealysin can lead to the disruption of FtsZ’s attachment to the membrane, and thereby inhibit bacterial division. Since the protealysin operon encodes not only the protease, but also its inhibitor, which is typical for the system of interbacterial competition, we assume that in the case of penetration of protealysin into neighboring bacteria that do not synthesize a protealysin inhibitor, cleavage of FtsZ and RecA by protealysin may give *S. proteamaculans* an advantage in interbacterial competition.

## 1. Introduction

The Gram-negative bacteria *Serratia* are facultative pathogens able to cause nosocomial infections or infections in immunocompromised patients [1]. *S. proteamaculans* can invade eukaryotic cells and this ability correlates with the activity of the bacterial actin-specific protease protealysin [2]. In vitro protealysin cleaves both globular and fibrillar actin, resulting in a restorable loss of the actin ability to polymerize [2,3,4]. In the host cell, protealysin can provide rearrangements of the actin cytoskeleton that is necessary for invasion. We have previously shown that protealysin penetrates into eukaryotic cells during co-incubation [3]. At the same time, protealysin is not secreted from bacteria into the extracellular environment [5], and a delivery system for bacterial effectors is required for protealysin penetration into eukaryotic cells. Delivery can be carried out both by bacterial outer membrane vesicles, as shown for grimelysin from *Serratia grimesii* [6], and by the bacterial secretion system. *S. proteamaculans* has a type VI secretion system (T6SS) that is widespread in Gram-negative bacteria [7]. The T6SS fires toxic proteins into target cells. The T6SS represents an efficient means by which bacteria interact with host organisms or attack competitors and may be triggered by contact from an attacking neighbor cell as a defensive strategy [8]. The same bacterial effector can act both in bacterial and eukaryotic cells. For example, the TplE phospholipase of *Pseudomonas aeruginosa* delivered through the type VI secretion system triggers the killing of bacterial competitors and promotes autophagy in epithelial cells [9]. The *Clostridium perfringens* iota toxin modifies eukaryotic actin [10], while the structurally related *S. proteamaculans* Tre1 toxin targets the bacterial cytoskeletal protein FtsZ [11]. These data point to an important role that bacterial virulence factors may play in the interbacterial antagonism.

Contact-dependent interbacterial competition is generally mediated by secreted toxic effector proteins. Therefore, bacteria require a means of inhibiting self-intoxication. Protection against effectors of these systems has been shown to derive from the production of specific immunity determinants that bind cognate effectors and inhibit their enzymatic function [12]. The loci coding for antibacterial effectors also harbor genes coding for the immunity proteins, and often both genes belong to the same operon [12]. This arrangement of protealysin and its inhibitor genes (emfourin) was found in *S. proteamaculans* [13]. It was shown by mass spectrometry that protealysin cleaves the bacterial outer membrane protein OmpX, which regulates the intensity of bacterial adhesion, as well as other minor bacterial proteins [14]. However, all these proteins are not critical for bacterial survival. Therefore, our work was aimed at searching for protealysin substrates among bacterial proteins that are responsible for bacterial survival in competing bacteria.

In this work, using purified genetically engineered proteins, we determined if the bacterial cell division protein FtsZ and the SOS response protein RecA are protealysin substrates. We have shown that in vitro protealysin cleaves the C-terminal segment of FtsZ from different microorganisms. The absence of the C-terminal fragment prevents FtsZ from attachment to the membrane and inhibits bacterial division [15]. In addition, we have shown that protealysin cleaves the SOS response protein RecA. Even 20% of the cleaved RecA in solution appears to be incorporated into the polymer of the uncleaved monomers, preventing further polymerization and inhibiting RecA ATPase activity. At the same time, the protealysin without its inhibitor reduces the survival of bacteria exposed to ultraviolet (UV) radiation. The data obtained in vitro were confirmed using genetically engineered bacteria in vivo. Therefore, in the case of protealysin penetration into neighboring bacteria that do not synthesize a protealysin inhibitor, the cleavage of FtsZ and RecA by protealysin may provide an advantage for *S. proteamaculans* in interbacterial competition.

## 2. Results

### 2.1. Protealysin Activity in OMVs

**Figure 1:**
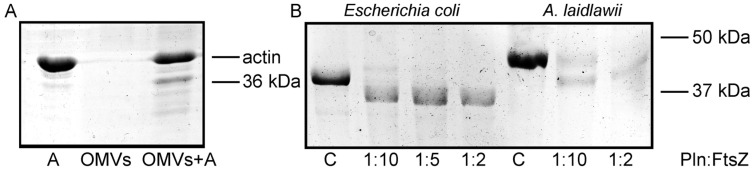
(**A**). The actin cleavage by the OMVs components. Purified OMVs were incubated with actin at the volume ratio of 1:1 for 18 h at 4 °C. A—control actin. (**B**). The FtsZ cleavage by protealysin in solution. Purified *E. coli* FtsZ and *A. laidlawii* FtsZ were incubated with protealysin in the mass ratio indicated below for 18 h at 4 °C. C—control (corresponding FtsZ, uncleaved).

Under poor nutrition conditions, removing organisms from a competitive environment can be critical for the survival of strains. In mixed population infections, outer membrane vesicles (OMVs) may aid strain survival by eliminating competing bacterial strains [16]. We have previously shown that *Serratia grimesii* OMVs carry the protease grimelysin [6]. In this work, we assessed whether *Serraria proteamaculans* OMVs carry the protease protealysin. Purified bacterial OMVs were incubated with actin. A 36 kDa fragment characteristic of protealysin cleavage appeared in solution during incubation (Figure 1A). Thus, OMVs can act as long-range protealysin delivery vectors to bacteria.

### 2.2. Cleavage of Purified FtsZ by Protealysin

FtsZ is an attractive target for bacterial effectors in competitor bacteria, and interference with its function results in rapid growth arrest and loss of viability [11]. Therefore, we determined whether FtsZ is a protealysin substrate. The *Serraria proteamaculans* FtsZ protein is 96% identical to the FtsZ from *E. coli* (Supplementary S1). We showed that protealysin limitedly cleaves purified *E. coli* FtsZ (Figure 1B). The FtsZ molecule consists of the N-terminal filament-forming globular domain and the C-terminal peptide that binds proteins at the bacterial membrane, connected by a 50-amino acid linker membrane [17,18]. The 320 amino acids of N-terminal domain are responsible for the polymers’ formation and have GTPase activity [15]. To determine the site of the proteolysis, the bands of the full-length and cleaved protein were excised from the gel (Figure 1B), digested with trypsin, and the intensities of the resulting peptides were compared by mass spectrometry (Supplementary S2). The intensities of the peaks corresponding to the found protein peptides were normalized to the sum of all the intensities of the spectrum peaks. The obtained values of the normalized intensities for each peptide were averaged over eight spectra. The final relative mean intensities of the peaks corresponding to the peptides of the uncleaved and proteolyzed FtsZ proteins were compared (Supplementary S3).

The average value of the intensities corresponding to the first peptide of the linker (320–329 a.a.) in the spectrum of the cleaved FtsZ turned out to be more than two times less than the corresponding one in the spectra of the untreated protein. The average relative intensity of the peaks corresponding to the next two linker peptides (330–338 a.a., 339–356 a.a.) was significantly less than the intensities of the corresponding peaks of the untreated protein (25 and 10 times, respectively). Moreover, the peaks corresponding to the next peptides (357–367 a.a., 368–379 a.a.) completely disappeared from the spectrum of the cleaved FtsZ (Supplementary S3). These data allow us to conclude that the N-terminal domain contains no cleavage sites available for proteolysis, while the linker has multiple cleavage sites, which is supported by the literature data (Supplementary S4) [19].

To determine whether differences in the FtsZ sequence could protect bacteria from protealysin cleavage, we tested whether the FtsZ from a different microorganism is a protealysin substrate. We chose *Acholeplasma laidlawii* FtsZ which is less than 51% identical to *S. proteamaculans* FtsZ (Supplementary S1). The protealysin also limitedly cleaved purified *A. laidlawii* FtsZ (Figure 1B). Using mass spectrometry, we showed that an *A. laidlawii* FtsZ N-terminal fragment remains uncleaved by protealysin (Supplementary S3). The peptide signal 328–334 a.a. in the cleaved FtsZ spectrum is six times less strong (averaged over six spectra of different intensities) compared to the untreated protein. In the spectrum of the cleaved protein, the signals corresponding to peptides after the 335th amino acid practically completely disappeared. Thus, protealysin cleaves the linker at several sites even in FtsZ, which has low homology to *S. proteamaculans* FtsZ (Supplementary S1).

### 2.3. Cleavage of FtsZ by Protealysin in Bacteria

**Figure 2:**
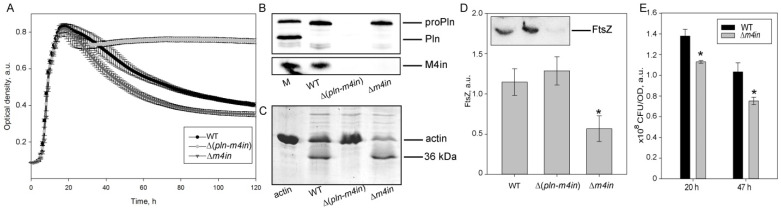
The FtsZ cleavage by protealysin in bacteria. (**A**). Growth curves of *S. proteamaculans* variants. Values are expressed as a mean of three repetitions ± S.D. (error bars). A difference was considered significant at the * *p* < 0.05 level. (**B**). Western blot detection of the expression of *pln* and *m4in* genes in *S. proteamaculans*. M—marker containing purified mature protealysin (Pln), protealysin precursor (proPln), and emfourin (M4in). WT, ∆(*pln-m4in*) and ∆*m4in* lanes were loaded with samples prepared from equal amounts of bacterial biomass. (**C**). Cleavage of actin by extracts of *S. proteamaculans* variants at 68 h of growth. (**D**). The number of FtsZ (estimated by Western blotting) in the same number of bacteria (determined by OD_600_) at 16 h of growth. Values are expressed as mean of three repetitions ± S.D. (error bars). A difference was considered significant at the * *p* < 0.05 level. The insert shows a representative blot. An analysis of three technical replicates is presented. WT—wild-type *S. proteamaculans*; ∆(*pln-m4in*)—mutant *S. proteamaculans* with in-frame deletion of both genes of the protealysin operon; ∆*m4in*—mutant *S. proteamaculans* with in-frame deletion of emfourin gene. (**E**). The number of bacteria (CFU) in the bacterial suspensions at 20 and 47 h of growth normalized to the OD600 is shown in the insert.

In *S. proteamaculans,* the protealysin gene (*pln*) and its inhibitor gene are colocalized within the single operon [13]. The protealysin can cleave its substrates only in the absence of its inhibitor, called emfourin (M4in). In order to determine whether in vivo protealysin can cleave FtsZ and suppress bacterial division, the *S. proteamaculans* with an in-frame deletion of the protealysin operon (*Δ(pln-m4in)*) or the emfourin gene (*Δm4in*) were constructed. Using a Western blot analysis, we confirmed that the Pln was absent in the Δ(pln-m4in) strain and the M4in was absent in the Δm4in and Δ(pln-m4in) strains (Figure 2B). The growth curve shows that *Δm4in* leads to an earlier decrease in optical density at 600 nm (OD_600_) when compared to wild-type *S. proteamaculans*, and the OD_600_ of *Δ(pln-m4in)* remains constant at the stationary phase of growth (Figure 2A). This may be associated with the cell death caused by the toxic effect of the protease. Deletion of the protealysin operon results in the loss of actinase activity in bacterial extracts (Figure 2C). During the incubation, the extract of the wild-type *S. proteamaculans* cleaved 40% of actin, and the extract of the *S. proteamaculans Δm4in* cleaved 65%. Using a Western blot with anti-FtsZ antibodies, we showed that deletion of the *m4in* gene reduces the amount of FtsZ in bacteria (Figure 2D). Reducing the amount of FtsZ can lead to disruption of the bacterial division apparatus. To test this idea, we determined the ratio of colony-forming unit (CFU) to the OD_600_ of the bacterial culture. Viable bacteria with an inactivated protealysin inhibitor of the same OD_600_ were 20–30% less abundant in suspension than wild-type bacteria, depending on the growth time (Figure 2E). This indicates that protealysin is toxic to bacteria, and/or the cleavage of FtsZ by protealysin causes a change in cell shape or increased chain formation after replication, as has been shown for *E. coli* transformed with the pProPlnHis_6_ plasmid [20].

### 2.4. Regulation of the RecA Activity and UV Survivability by Protealysin 

**Figure 3:**
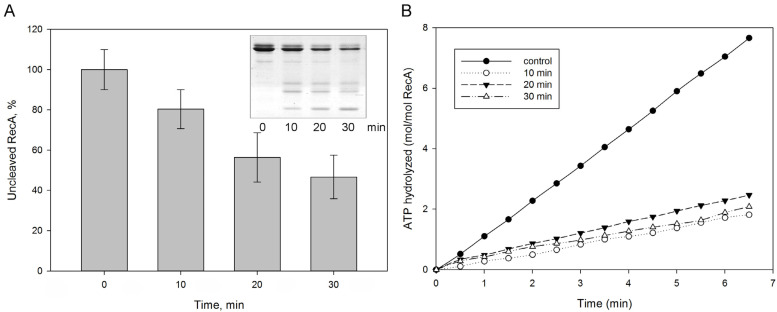
The RecA cleavage by protealysin in solution. Purified *E. coli* RecA was incubated with protealysin in a Pln/RecA mass ratio of 1:20 for 10, 20 and 30 min at 22 °C. (**A**). The amount of uncleaved RecA was determined by electrophoregram (insert). (**B**). ATPase activity of RecA after 10, 20, 30 min incubations with protealysin.

We also assessed whether protealysin could affect bacterial survival under severe stress conditions. When DNA is damaged, the SOS response is triggered [21]. The RecA protein plays a key role in triggering the SOS response [22]. RecA forms an ATP-dependent filament around single-stranded DNA regions that accumulates as a result of DNA damage [22]. The RecA protein in solution forms structures of various shapes from several monomers. Therefore, it is impossible to evaluate its polymerization using the standard method for measuring light scattering. To assess polymerization, we used the measurement of the amount of ATPase activity due to polymerization, accompanied by ATP hydrolysis. We have shown that protealysin cleaves the purified SOS response protein RecA unlimitedly and quantified the amount of cleaved RecA by electropherogram (Figure 3A). Surprisingly, 20% of the digested protein in solution is sufficient to inhibit the RecA ATPase activity (Figure 3B). Apparently, fragments of the cleaved RecA binding to the polymer prevent the further addition of monomers.

**Figure 4:**
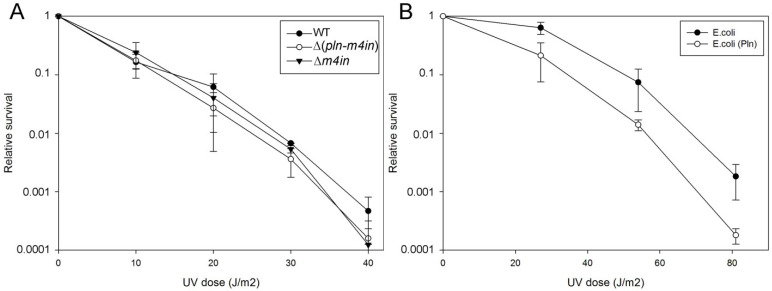
UV survivability of bacteria (**A**). UV survivability of wild-type *S. proteamaculans* (WT, black circles), and mutant *S. proteamaculans* with an in-frame deletion of both genes of the protealysin operon (∆(*pln-m4in*), open circles) and with an in-frame deletion of the emfourin gene (∆*m4in*, black triangles). (**B**). UV survivability of *E. coli* AB1157 transformed with the control plasmid (black circles) and the plasmid carrying the protealysin gene (open circles).

The SOS response is activated in response to DNA damage caused by UV radiation or chemical agents [21]. We have previously shown that in the absence of the *recA* gene, the first seconds of UV exposure lead to a lethal effect on *E. coli* before the activation of the SOS response [23]. However, we did not know if these effects are the same for a recA mutant in *S. proteoamaculans*. We compared the UV sensitivity of wild-type *S. proteamaculans* and mutant *S. proteamaculans* with in-frame deletions of both genes of the protealysin operon (∆(*pln-m4in*)) or of the emfourin gene (∆*m4in*). It turned out that deletion of the genes on the protealysin operon did not significantly affect UV sensitivity (Figure 4A). It is possible that under UV exposure RecA does not play a critical role for the *S. proteamaculans* survival, in contrast to the *E. coli* survival. To determine UV sensitivity, bacteria were plated on LB agar. The method does not allow for evaluating the activity of protealysin in bacteria on the plates. Previously, we showed that the quorum-sensing system responsible for sensitivity to population density is a regulator of protealysin activity in *S. proteamaculans* [24]. Thus, we cannot rule out that protealysin is not active when bacteria are plated. Therefore, we assessed whether protealysin in the bacterial cell could reduce the survival of UV-exposed bacteria in *E. coli* AB1157. Transformation of bacteria with a plasmid carrying the *pln* gene does not affect the viability of bacteria in the absence of UV exposure. This transformation reduced their survival by three times at a UV dose of up to 30 J/m^2^, and by ten times at a UV dose of more than 40 J/m^2^ (Figure 4B). Thus, in bacteria that do not synthesize proteins of the emfourin family, protealysin may suppress the bacterial SOS response by cleaving the RecA protein, which leads to a decrease in the bacterial survival.

## 3. Discussion

The protease protealysin is a *S. proteamaculans* virulence factor. Its substrate is actin and proteins involved in invasion [2,14]. This work was aimed at finding the protealysin substrates essential for bacterial survival. We have shown that protealysin can cleave the RecA protein *in vitro*. Even 20% of the cleaved protein in solution prevents the ATP hydrolysis of RecA filaments. RecA is one of the most important factors for bacterial survival under stress conditions [21]. On the other hand, RecA may play a role in the regulation of bacterial adhesion and invasion. In order to avoid host immunity, a continuous change in the adhesion-responsible pilus is necessary. In a RecA-deficient strain, pilus phase variation occurs at a 100–1000-fold reduced rate [25]. RecA-dependent pathways mediate the binding of *Staphylococcus aureus* with fibronectin extracellular matrix protein [26] and regulates the type III secretion system of enteropathogenic *E. coli* [27]. Furthermore, activation of *Listeria monocytogenes* recA was shown during the adhesion and invasion of Caco-2 intestinal epithelial cells [28]. Moreover, an in-frame RecA deletion mutant showed a deficiency in the adhesion to and invasion of Caco-2 cells [28]. Thus, cleavage of RecA in bacteria can reduce their virulence. We have previously shown that inactivation of the protealysin gene increases the number of *S. proteamaculans* that penetrated into the host cell during co-incubation [14]. This indicates that protealysin is not completely inhibited in the wild strain of *S. proteamaculans*. Apparently, the accumulation of uncleaved RecA in these bacteria may be a regulator of bacterial invasion. 

FtsZ is a bacterial tubulin homologue essential for cell division in almost all bacteria [29]. The FtsZ molecules assemble into filaments, which further associate to make the Z ring. FtsZ’s C-terminal, a 15–17 amino acid peptide, binds FtsA and ZipA, membrane-anchored proteins that are essential components of the Z ring structure that mediates cell division [17,18]. FtsZ has a 50-amino acid linker between the filament-forming globular domain and the C-terminal peptide that binds FtsA and ZipA. This linker is widely divergent across bacterial species and thought to be an intrinsically disordered peptide [30]. The FtsZ linker has long been thought to function as a flexible tether connecting the FtsZ filaments to the cell membrane, and any intrinsically disordered peptide of appropriate length would be able to substitute for the linker in *E. coli* FtsZ [30]. However, according to our results, this linker may contain sites for cleavage by regulatory proteases. 

Previously it was shown that the 320-amino acid, truncated N-terminal of FtsZ is a potent inhibitor of cell division [15]. This inhibitory activity is likely to arise from the truncated FtsZ copolymerizing with a full-length FtsZ. Although the truncated FtsZ is able to form polymers and has GTPase activity, the copolymers formed between FtsZ and the truncated FtsZ are not capable of functioning as a Z ring [15]. Additionally, the truncated FtsZ does not interact with FtsA [15]. Thus, cleavage of the C-terminal segment by protealysin can defect FtsZ attachment to the membrane and inhibition of bacterial division. 

According to our data, protealysin may be a regulator of bacterial division and bacterial SOS responses. We have previously shown the colocalization of protealysin gene with the gene encoding the inhibitor of protealysin and thermolysin called emfourin. Emfourin forms a complex with protealysin with a 1:1 stoichiometry, and binds the active site region [13]. The protealysin and emfourin genes belong to the same operon [13]. This arrangement of the genes for the enzyme and its inhibitor is characteristic of the genes related to antibacterial toxins and bacterial immunity proteins [12]. In this case, protection against antibacterial effectors occurs due to the production of specific determinants of immunity that bind related enzymes and inhibit their enzymatic function [7]. This suggests that protealysin may be a toxic effector of interbacterial competition systems where emfourin-like inhibitors function as immunity proteins, protecting sister cells. Therefore, in the case of protealysin delivery into neighboring bacteria that do not synthesize emfourin, cleavage of FtsZ and RecA by protealysin may inhibit bacterial division and bacterial SOS responses, which will provide an advantage for *S. proteamaculans* in interbacterial competition.

## 4. Materials and Methods

### 4.1. Strains and Plasmids

The *Escherichia coli* strains TG1 and CC118 λ pir were used for plasmid maintenance. The *E. coli* ST18 strain [31] was obtained from the German Collection of Microorganisms and Cell Cultures (DSM 22074) and used for conjugal transfer of plasmids into *S. proteamaculans* 94 [32]. *E. coli* and *S. proteamaculans* strains were grown on Luria broth (LB) supplemented with 100 µg/mL ampicillin, 50 μg/mL kanamycin or 100 µg/mL streptomycin as needed. When *E. coli* ST18 was cultured, the medium contained 50 μg/mL 5-aminolevulinic acid (Sigma Aldrich, Darmstadt, Germany). To plot *S. proteamaculans* strains’ growth curves, overnight cultures were diluted with LB to OD_600_ of 0.01. The bacterial suspensions (100 μL) were transferred to a 96-well plate and cultivated at 30 °C and 200 rpm using a microplate reader, Infinite M200 Pro (Tecan, Switzerland). The optical density was read at a wavelength of 600 nm. *E. coli* AB1157 (*thr-1 leuB6 ara14 proA2 hisG4 argE3 thi-1 supE44 rpsL31*) from R. Devoret’s collection was co-transformed with pProPlnHis_6_ plasmid, which contains the protealysin gene under control of the T7 promoter [20,33] and the pT7POL26 construct for T7 promoter-driven protein expression from Cox M.M. The presence of both plasmids was controlled by adding a mixture of ampicillin and kanamycin.

### 4.2. Generation of In-Frame Deletion Mutant Strains of S. proteamaculans

For the production of deletion constructs, regions (~500 bp) flanking the deletion were amplified using the pSP1.8 plasmid, which contained a 3.3 kb *S. proteamaculans* DNA segment with the *pln* gene [32] as a template, which was joined by overlap extension PCR, and ligated into the *Aat* II and *Sac* I sites of the pAL2-T vector (Evrogen, Russia). The primer pairs op_combR/pln_Aat and m4in_Sac/op_combD were used to construct the *S. proteamaculans* variant with a deletion of both genes of the protealysin operon (*Δ(pln-m4in*)); the m4in_Sac/m4in_combD and m4in_Aat/m4in_combR pairs were used for the variant with an in-frame deletion of only the emfourin gene (*Δm4in*). All the primers used in plasmid construction and generation of mutant strains are listed in Table 1. The cloned fragments were transferred into *Xba* I and *Sac* I sites of the suicide vectors pRE118, which were a gift from Dieter Schifferli (Addgene plasmid #43830) [34]. The structure of cloned fragments was confirmed by Sanger sequencing.

To generate deletions of *S. proteamaculans* strains, deletion constructs in pRE118 were transformed into *E. coli* ST18. The constructs were transferred from donor *E. coli* ST18 into recipient *S. proteamaculans* 94 by conjugation as described in [35]. Briefly, *E. coli* ST18 donor strains carrying the mutant constructs and the recipient *S. proteamaculans* 94 were grown overnight in LB with aeration at 37 and 30 °C, respectively. Overnight cultures (50 µL) were used to inoculate 5 mL of fresh LB, which were incubated under the same conditions to mid-log phase and concentrated by centrifugation at 8500× *g* for 1 min. Concentrated donor and recipient bacteria were mixed at a ratio of 3:1 (based on OD_600_) and placed on a sterile cellulose acetate filter with a pore size of 0.45 µm (Sartorius) on an LB agar plate (the medium did not contain 5-aminolevulinic acid). After overnight incubation at 30 °C, the bacterial mixture was washed off the filter and resuspended in LB. The resuspended bacteria mixture was spread on LB agar containing 50 μg/mL kanamycin to select *S. proteamaculans* transconjugants. Isolated colonies were propagated in LB containing 50 μg/mL kanamycin and incubated with shaking at 30 °C overnight. Then, 10 µL of culture was diluted in 1 mL of LB and 100 µL of the resulting suspension was spread on an LB (without NaCl) agar plate with 10% sucrose to select for the *S. proteamaculans* containing the desired mutation. Sucrose-resistant and kanamycin-sensitive colonies were screened for allelic replacement by colony PCR using op_exD/op_exR and op_inD/op_inR primer pairs for the Δ(*pln-m4in*) variant, as well as m4in_exD/op_exR and m4in_inD/op_inR pairs for the Δ*m4in* mutant.

### 4.3. Protein Purification

FtsZ from *E. coli* was cloned and purified as described in [36], with modifications. The pET-21a vector was used instead of pET-11. Both 20 and 25% of ammonium sulfate as final concentrations were used, and no difference between fractions of FtsZ was found. For FtsZ from *Acholeplasma laidlawii,* 50% of ammonium sulfate was used as the final concentration during precipitation. FtsZ in TNEM buffer (100 mM NaCl, 1 mM EDTA, 5 mM MgCl_2_, 50 mM Tris–HCl, pH = 8.0) was precleared by centrifugation at 16,000× *g* for 10 min and further loaded on a size-exclusion column (GE HiLoad^®^ 16/600 Superdex^®^ 200 pg). The column was preliminarily equilibrated with the same buffer. The chromatographic purification was implemented on an Akta Purifier 10 (GE Healthcare) with a flow rate of 0.6 mL/min. The protein fractions that passed through the column and analyzed by UV detector (wavelength of 280 nm) were collected (each fraction—2 mL) and further analyzed by SDS-PAGE electrophoresis using the standard Laemmli protocol with 10% acrylamide gel. FtsZ concentration was determined by the Bradford assay. The *E. coli* RecA protein was overexpressed and purified as described previously [37]. The concentration of the purified protein was determined from the absorbance at 280 nm using the extinction coefficient 2.23 × 10^4^ M^−1^ cm^−1^ [38]. 

### 4.4. OMVs Isolation 

*S. proteamaculans* 94 colonies were grown in LB medium (Sigma Aldrich, Darmstadt, Germany) at 30 °C in aerated flasks for 41 h. Outer membrane vesicle (OMV) formation was induced by adding hydrogen peroxide to the medium until a final concentration of 250 μM (Biolot, St. Petersburg, Russia) for 1 h; after this, a OMV isolation was performed. The bacteria were centrifuged at 3160× *g* for 25 min, the supernatant was additionally clarified with two repeated centrifugation cycles and the resulting supernatant was filtered through a membrane with a pore diameter of 0.22 μm (Millipore, Watford, UK). The supernatant was subjected to ultracentrifugation at 40,000× *g* for 1 h at 4 °C using a Type 45 Ti rotor (Beckman Instruments, Indianapolis, IN, USA). Sediments containing OMVs were suspended in PBS (Biolot, St. Petersburg, Russia). The OMV suspension was tested for sterility by plating on LB agar plates (Sigma Aldrich, Darmstadt, Germany). 

### 4.5. Determination of Protealysin Substrates

Purified RecA was mixed with protealysin purified as described previously [33]. Purified *E. coli* RecA was incubated with protealysin at a Pln/RecA mass ratio of 1:20 for 10, 20 and 30 min at 22 °C. The reaction was stopped by 5 mM 1,10-phenanthroline. 

Purified *E. coli* FtsZ and *A. laidlawii* FtsZ were incubated with protealysin for 18 h at 4 °C. The reaction was stopped by the addition of an equal volume of the electrophoresis sample buffer containing 4% SDS, 125 mM Tris–HCl, pH 6.8, followed by 5 min of boiling. The digestion products were analyzed by SDS-PAGE [39]. The protein bands of the native and truncated proteins were excised from the gel and analyzed by mass spectrometry. 

### 4.6. Sample Preparation and MS Analysis

The gel fragments containing specific bands were minced and treated twice with 60 mM NH_4_HCO_3_ in 40% acetonitrile (ACN) for 20 min at 37 °C in a shaker for destaining. After drying, the gel pieces with 100% ACN they were rehydrated in 50mM NH_4_HCO_3_ and 10% ACN containing 15 μg/mL proteomics grade trypsin (Sigma Aldrich, Darmstadt, Germany), and were then incubated for 2 h on ice and 4 h at 37 °C. Supernatants were collected and 0.5 μL of protein fragments solutions were spotted on a steel plate with 0.5 μL of 2.5-Dihydroxybenzoic acid matrix (Sigma Aldrich, Darmstadt, Germany; 20 mg/mL in 50% aqueous acetonitrile, 0.1% TFA) and dried at room temperature [14]. High resolution mass spectra were recorded on a Fourier Transform Ion Cyclotron Resonance Mass spectrometer (FT-ICR MS, Varian 902-MS, Palo Alto, CA, USA) equipped with a 9.4 Tesla superconducting magnet in the positive MALDI (matrix-assisted laser desorption/ionization) mode. A ProteoMass Peptide MALDI-MS Calibration Kit (Sigma Aldrich, Darmstadt, Germany) was used for external calibration. For internal mass calibration, the residual trypsin peak (842.50940 Da) was used. Mass spectrometry data analysis was carried out using FTDocViewer software (Varian) and proteins were identified using a Mascot peptide mass fingerprint software program (www.matrixscience.com, accessed on 8 August 2022). Mass spectra were searched against protein sequences from the NCBIprot knowledge database using the Bacteria search engine. The initial search parameters allowed for a mass error of up to ±5 ppm, a single trypsin-missed cleavage and possible methionine oxidation. Besides this, the Protein Prospector MS-Fit software was used to confirm the protein sequence and search for the proteolysis site (http://prospector.ucsf.edu, accessed on 8 August 2022).

### 4.7. Actinase Activity

Rabbit skeletal muscle actin used as a substrate for protealysin was isolated by a standard procedure of Spudich and Watt [40]. G-actin in buffer G (0.2 mM ATP, 0.1 mM CaCl_2_, 5 mM Tris–HCl, pH 7.5, 0.02% NaN_3_) was stored as aliquots (1–3 mg/mL) at −20 °C for a single use. *S. proteamaculans* colonies were grown in LB at 30 °C with aeration until the late stationary growth phase until actinase activity in the *S. proteamaculans* extract could be determined [1]. To determine the ability of bacterial extracts to cleave actin, bacteria were pelleted by centrifugation at 12,000× *g* for 10 min, the pellets were re-suspended in buffer G, and the bacteria were lysed using seven cycles of freeze and thaw. The bacterial lysates were clarified by centrifugation at 12,000× *g* for 10 min. The clarified bacterial lysates was mixed with an equal volume of actin and incubated for 18 h at 4 °C. The reaction was stopped by the addition of an equal volume of the electrophoresis sample buffer containing 4% SDS, 125 mM Tris–HCl, pH 6.8, followed by 5 min of boiling. The digestion products were analyzed by SDS-PAGE [39]. The actinase activity was determined by the appearance of the 36 kDa actin fragment. 

### 4.8. Western Blot Analysis

Wild-type, Δ(*pln-m4in*) and Δ*m4in* strains were grown in LB for 16 h at 30 C with aeration until the stationary phase. This growth time was chosen because protealysin is inactive at the exponential stage of growth [2], and the amount of FtsZ decreases with further growth. Bacteria were pelleted by centrifugation at 4200× *g* for 30 min, the pellets were re-suspended in lysis buffer (200 μg/mL lysozyme, 5 mM 1,10-phenanthroline, 0.1% triton in PBS) and the bacteria were lysed using seven cycles of freeze and thaw. The lysates were clarified by centrifugation at 12,000× *g* for 10 min. The clarified bacterial lysates were boiled with an electrophoresis sample buffer (4% SDS, 24% glycerol, 200 mM DTT, 0.01% bromphenol blue, 125 mM Tris–HCl, pH 6.8) for 5 min. 

The samples were fractionated by SDS-PAGE and transferred to a Hybond ECL membrane according to the manufacturer’s instructions (GE Healthcare, Buckinghamshire, UK). The membrane was incubated with 5% BSA in PBS 40 min to prevent the nonspecific binding of antibodies and then incubated with rabbit primary antibodies against FtsZ at a dilution of 1: 500 (Agrisera, Vännäs, Swedish) at room temperature for 2 h. The membrane was then washed three times with a washing buffer (5% BSA, 0.1% Tween 20, PBS) for 10 min and incubated for 2 h with the secondary antibodies (1:20,000) against rabbit IgG, which were conjugated with horseradish peroxidase. The membranes were washed with a washing buffer three times and developed using SuperSignal West FEMTO Chemiluminescent Substrate (ThermoFisher Scientific, Waltham, MA, USA) according to the manufacturer’s recommendations.

The analysis of protealysin and M4in accumulation in *S. proteamaculans* cells was performed as described elsewhere [5,13].

### 4.9. ssDNA-Dependent ATP Hydrolysis (ATPase) Assays

A coupled enzyme spectrophotometric assay was used to measure RecA-mediated ATP hydrolysis. Purified *E. coli* RecA was incubated with protealysin at a Pln/RecA mass ratio of 1:20 for 10, 20 and 30 min at 22 °C. The reaction was stopped by 5 mM 1,10-phenanthroline. The ADP generated by hydrolysis was converted back to ATP by a regeneration system of pyruvate kinase and phosphoenolpyruvate (PEP). The presented system was designed to assess the hydrolysis of ATP molecules. The resultant pyruvate was converted to lactate by lactate dehydrogenase using NADH as a reducing agent. The hydrolysis of one ATP molecule results in the oxidation of one equivalent of NADH to NAD+. The conversion of NADH to NAD^+^ was monitored by a decrease in the absorbance at 380 nm [41]. ssDNA-dependent ATP hydrolysis reactions were carried out at 37 °C as described previously [42]. We used poly Poly(dT) (Sigma Aldrich, Darmstadt, Germany) as ssDNA. In the absence of RecA or ssDNA, there is no ATP hydrolysis in solution. Previously, we studied the biochemical properties of protealysin and showed that the protease itself does not have ATPase activity [3]. To control for the integrity of the ATPase detection system in the presence of inhibited protealysin, uncleaved RecA was added to the solution after measurement. The presence of inhibited protealysin did not affect ATPase activity.

### 4.10. UV Radiation Sensitivity

Bacterial cells grown in LB media with 100 mg/L ampicillin for OD_600_ = 0.4–0.6. 100 μL of appropriate dilutions were spread onto LB plates. Preliminary experiments with serial dilutions of samples by 10 times determined the necessary dilution of bacterial suspensions in the experiment. Samples that were diluted to 50 to 300 CFU were plated. The plates were then exposed to ultraviolet (UV) light in a calibrated Chromoscope UV at doses from 10 to 80 J/m^2^. After incubating at 37 °C overnight, the colonies were counted and divided by the dilution factor to obtain the colony-forming unit (CFU)/mL. For percentage of survival, colony counts on the treated plates were divided by the counts on untreated plates.

### 4.11. Statistical Analysis

For all experiments, at least three biological replicates were analyzed. The figures show the data of three technical repetitions. Data were analyzed using a one-way analysis of variance (ANOVA) with the Excel Data Analysis Pack. A difference was considered significant at *p*-values < 0.05.

## Figures and Tables

**Table 1:** Primers used.

Primer	Sequence *
pln_Aat	GACTGACGTCT**TCTAGA**CGGCTTCGGCAGCGAA
m4in_Sac	AACT***GAGCTC***GCTGTCCTCTTCGGCCAAGTTGT
op_combD	CTATGCCGGATGAGTGAACACTTACA
op_combR	TTCACTCATCCGGCATAGTGGCTCTCC
m4in_Aat	GACTGACG**TC****TAGA**TTTACTTCCAGCAAGC
m4in_combD	TGGCATAAACACTTACAACGGCTGATAAC
m4in_combR	GTTGTAAGTGTTTATGCCACCCCCACCTGAT
op_exR	GATTATTGGTCTGGTGTGCG
op_exD	AACATATTCACCAGTTAGCCG
m4in_exD	AGGCTTTCAAACGCAACTCGC
op_inR	TTGGGCATCTATTTCTTACG
op_inD	ACGAACTGGGCTATGAGG
m4in_inD	ATGAATCGCTGTCTGACG

* The *Aat* II sites are underlined, the *Xba* I sites are boldfaced, the *Sac* I site is italicized and boldfaced. The overlapping sequences are underlined with a double or wavy line. All primers were obtained from Evrogen (Russia).

## Supplementary Materials

The supporting information can be downloaded at: https://www.mdpi.com/article/10.3390/ijms231810787/s1. Reference [19] is cited in the supplementary materials.
