# Supplementary material for: Protealysin Targets the Bacterial Housekeeping Proteins FtsZ and RecA
Source: Int J Mol Sci. 2022 Sep 15;23(18):10787. doi: 10.3390/ijms231810787 (PMC9505478; doi:10.3390/ijms231810787)
Supplement: Supplementary file 1 [file ijms-23-10787-s001.zip › ijms-1883474-supplementary.pdf]

Supplementary S1. Alignment of the FtsZ amino acid sequence.

```
                 1       10        20        30        40        50        60        70        80        90       100
                 |--------+---------+---------+---------+---------+---------+---------+---------+---------+---------|
S.proteamaculans   MFEPMELTNDAVIKVIGVGGGGGNAVEHMVRERIEGVEFFAVNTDAQALRKTAVGQTIQIGSGITKGLGAGANPEVGRNSAEEDREALRAALDGADMV
          E.coli   MFEPMELTNDAVIKVIGVGGGGGNAVEHMVRERIEGVEFFAVNTDAQALRKTAVGQTIQIGSGITKGLGAGANPEVGRNAADEDRDALRAALEGADMV
     A.laidlawii MVFGFNDEFNQKPVIKVIGVGGGGNSAVNRMIENDVRGVSYVAMNTDAQVLKVSKADERIQLGKKLTRGLGAGAKPAIGKQAALESEDDIREVLSDADMV
       Consensus ..mfepmElt#daVIKVIGVGGGGgnAV#hM!r#r!eGVe%fAvNTDAQaLrktavg#tIQiGsgiTkGLGAGAnPe!Gr#AAlEdrDalRaaLsgADMV

                 101    110       120       130       140       150       160       170       180       190       200
                 |--------+---------+---------+---------+---------+---------+---------+---------+---------+---------|
S.proteamaculans FIAAGMGGGTGTGAAPVVAEVAKDLGILTVAVVTKPFNFEGKKRMAFAEQGIAELSKHVDSLITIPNDKLLKVLGRGISLLDAFGAANDVLKGAVQGIAE
          E.coli FIAAGMGGGTGTGAAPVVAEVAKDLGILTVAVVTKPFNFEGKKRMAFAEQGITELSKHVDSLITIPNDKLLKVLGRGISLLDAFGAANDVLKGAVQGIAE
     A.laidlawii FITAGMGGGTGTGAAPVVARIAKELGVLTIGIVTKPFVFEGPLRMQHAITGLEELKPNVDTLIVIPNERLFSIADRDMQLLDAFRESDKVLRQGVQGIAE
       Consensus FIaAGMGGGTGTGAAPVVAe!AK#LG!LT!a!VTKPFnFEGkkRMafAeqGieELskhVDsLItIPN#kLlk!lgRgisLLDAFgaa#dVLkgaVQGIAE

                 201    210       220       230       240       250       260       270       280       290       300
                 |--------+---------+---------+---------+---------+---------+---------+---------+---------+---------|
S.proteamaculans LITRPGLMNVDFADVRTVMSEMGYAMMGSGVACGEDRAEEAAEMAISSPLLEDIDLSGARGVLVNITAGFDLRLDEFETVGNTIRAFASDNATVVIGTSL
          E.coli LITRPGLMNVDFADVRTVMSEMGYAMMGSGVASGEDRAEEAAEMAISSPLLEDIDLSGARGVLVNITAGFDLRLDEFETVGNTIRAFASDNATVVIGTSL
     A.laidlawii IIAVPGMINVDFADVRTVMENKGTALMGIGMASGENRAIEAARKAIHSKLLE-VSIDGATDAIVNISSGAEVTLFEIEAALTEIRNATESDLNVIYGHTV
       Consensus lItrPG$mNVDFADVRTVMs#mGyA$MGsGvASGE#RAeEAAemAIsSpLLEd!dlsGArgvlVNItaGf#lrLdEfEtvgntIRafasd#atV!iGtsl

                 301    310       320       330       340       350       360       370       380     388
                 |--------+---------+---------+---------+---------+---------+---------+---------+-------|
S.proteamaculans DPEMNDELRVTVVATGIGMD-KRPEITLVTNKQA-SQPVMDHRYQQHGMSPLPQEVKPAAKVVNDPTAQPNKEPDYLDIPAFLRKQAD
          E.coli DPDMNDELRVTVVATGIGMD-KRPEITLVTNKQV-QQPVMD-RYQQHGMAPLTQEQKPVAKVVNDNAPQTAKEPDYLDIPAFLRKQAD
     A.laidlawii SVDLEDEMIVTIVATGYELRAKGNEVEKIAGDIFRNNSTQQVKITDTGLEPLNNKEASGEDTKKRTLPSWLHRK
       Consensus dpD$#DE$rVT!VATGig$d.KrpE!tl!tnkqf.##pvm#vryq#hG$ePLn#e#kpgakvvndtlPqwlkepdyldipaflrkqad
```

High consensus (60%) is highlighted in red. Low consensus (30%) is highlighted in blue. Neutral is highlighted in black. Consensus symbols: “!” = IV, “$” = LM, “%” = FY, “#” = NDQEBZ. The Multalin version 5.4.1 was used to build a consensus sequence.

Supplementary S2. Examples of mass spectra.

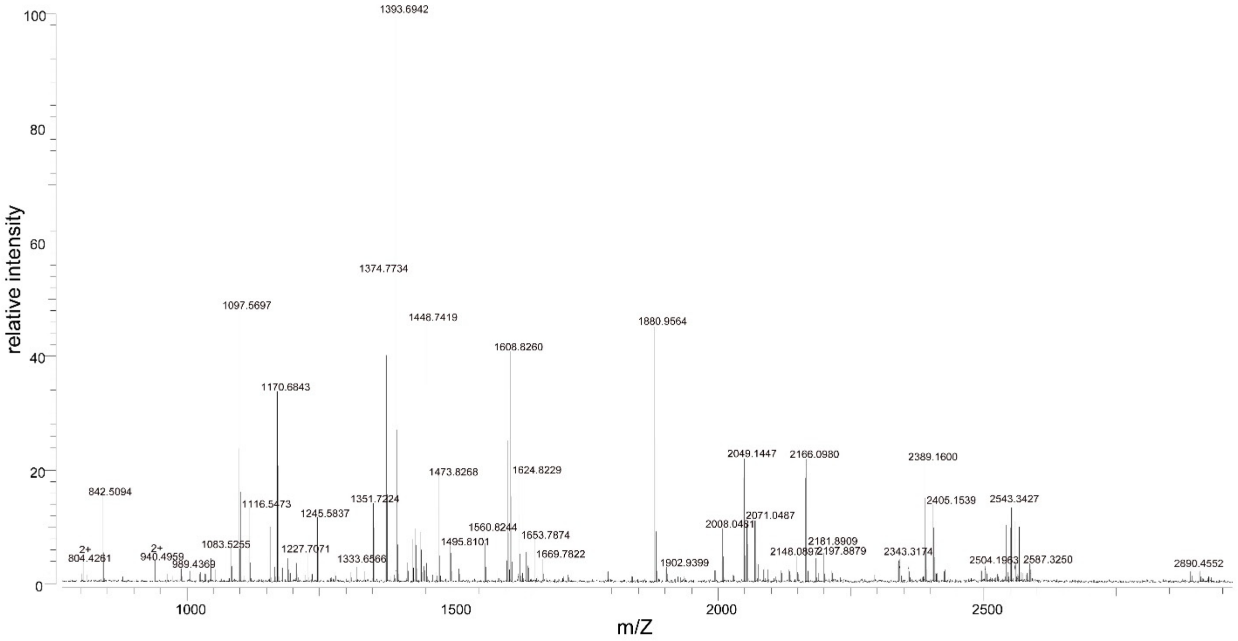

Spectrum of uncleaved *E. coli* FtsZ.

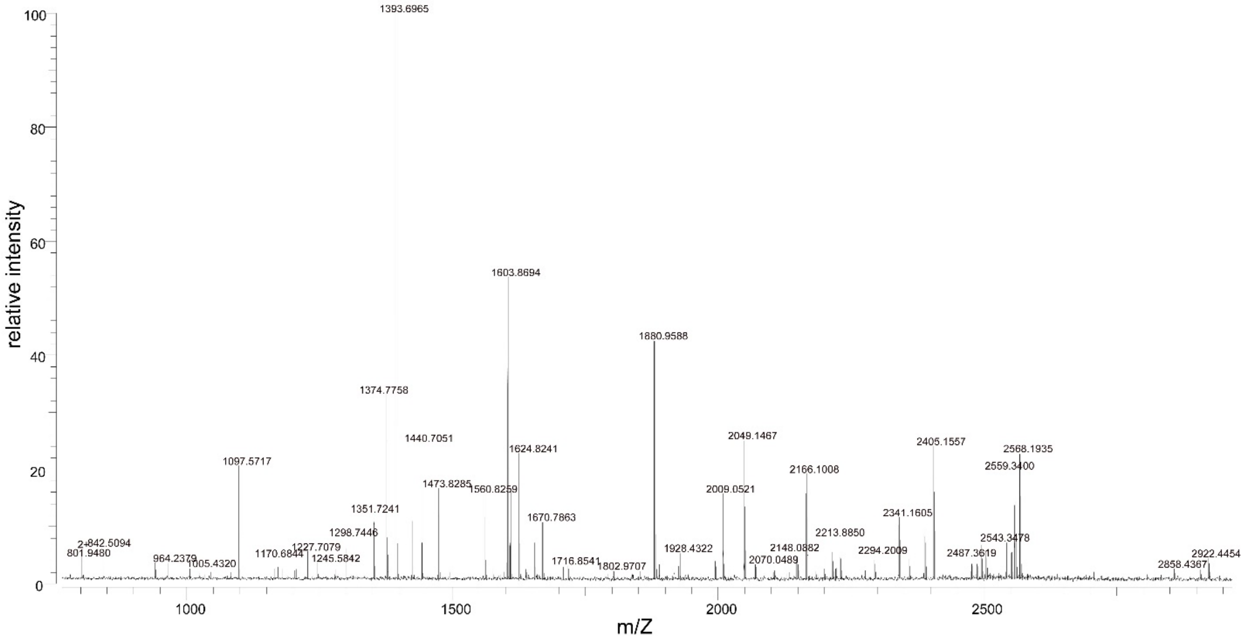

Spectrum of *E. coli* FtsZ cleaved by a protealysin.
Full mass range.

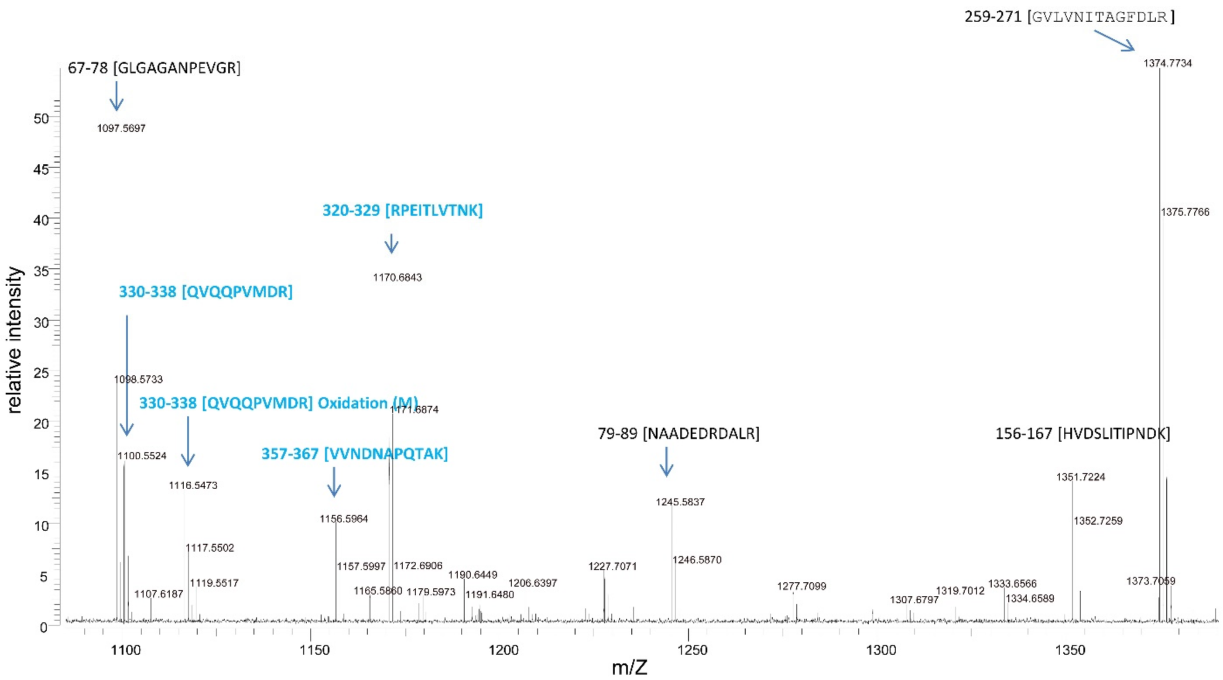

Spectrum of uncleaved *E. coli* FtsZ.

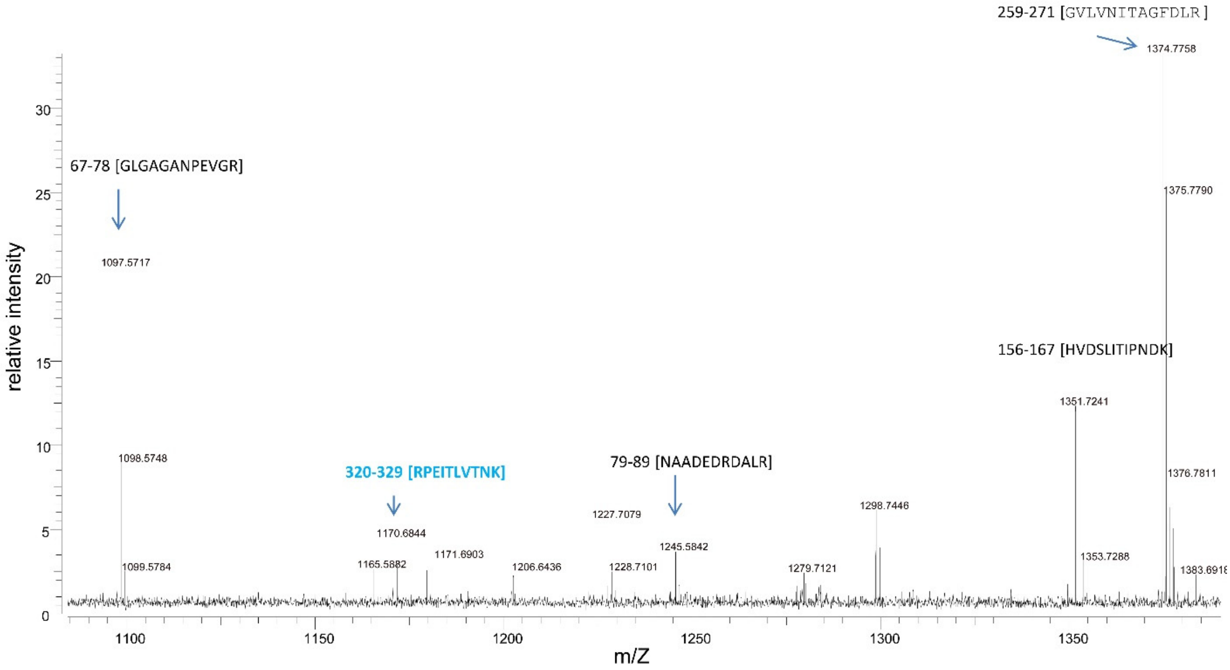

Spectrum of *E. coli* FtsZ cleaved by a protealysin.

The spectra are shown with the selected mass range, in which the peaks of the cleaved protein disappear from the spectrum or the intensity of which is greatly reduced. Their corresponding peptides are marked in blue.

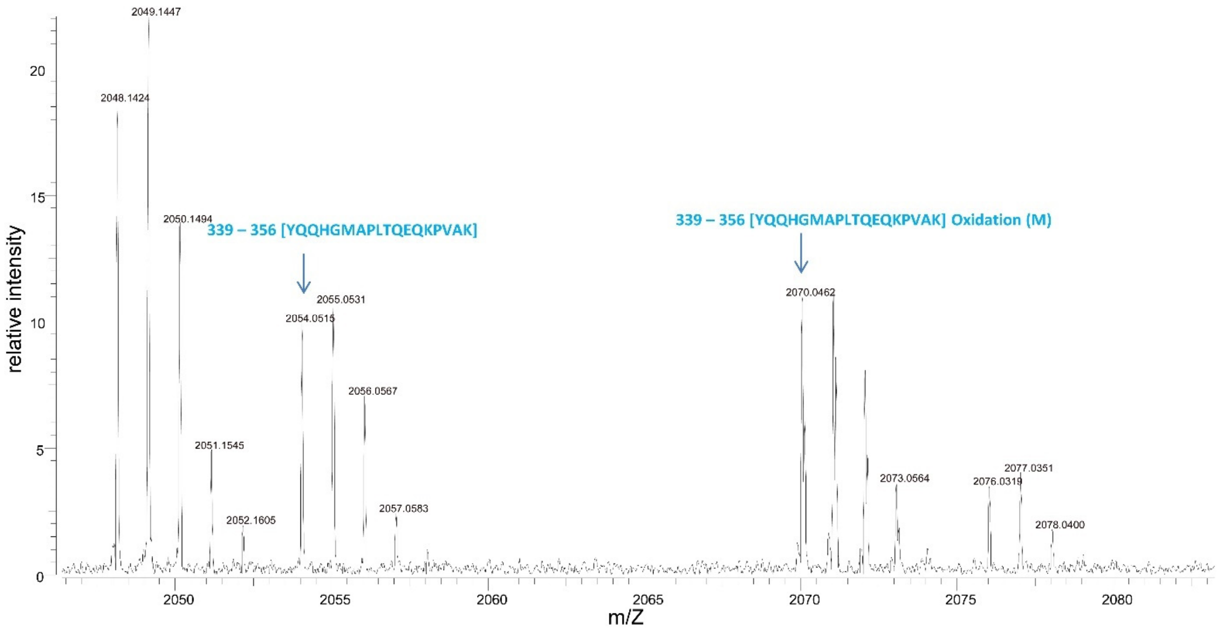

Spectrum of uncleaved *E. coli* FtsZ.

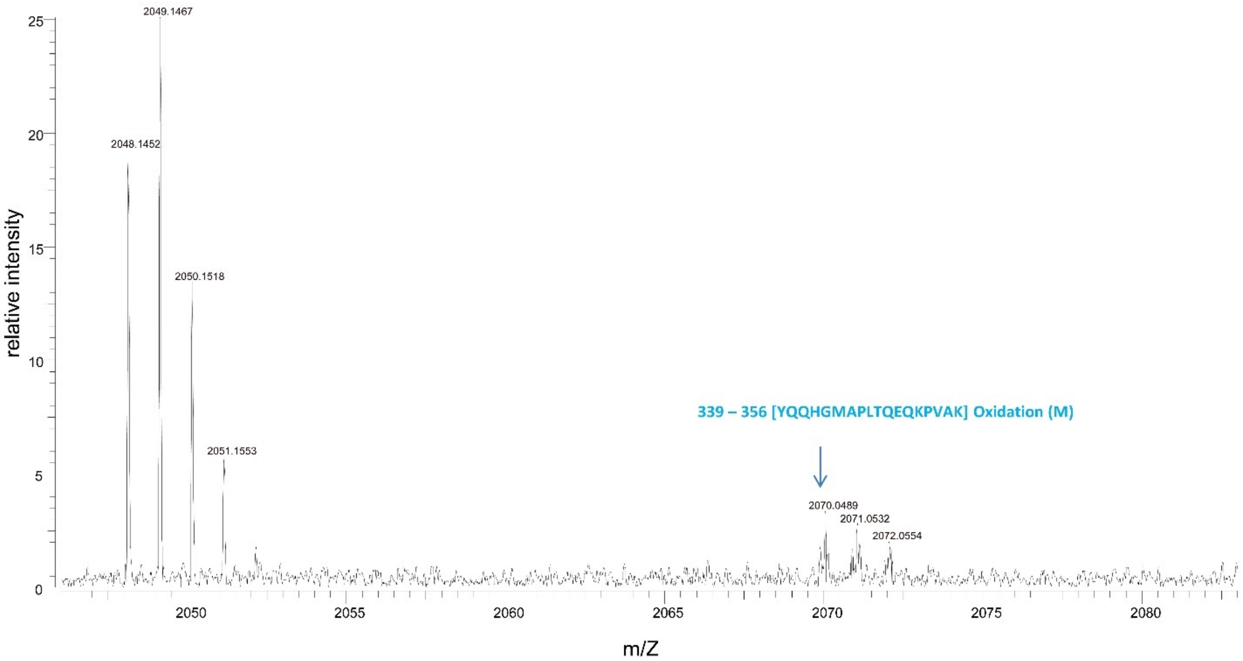

Spectrum of *E. coli* FtsZ cleaved by a protealysin.

The spectra are shown with the selected mass range, in which the peaks of the cleaved protein disappear from the spectrum or the intensity of which is greatly reduced. Their corresponding peptides are marked in blue.

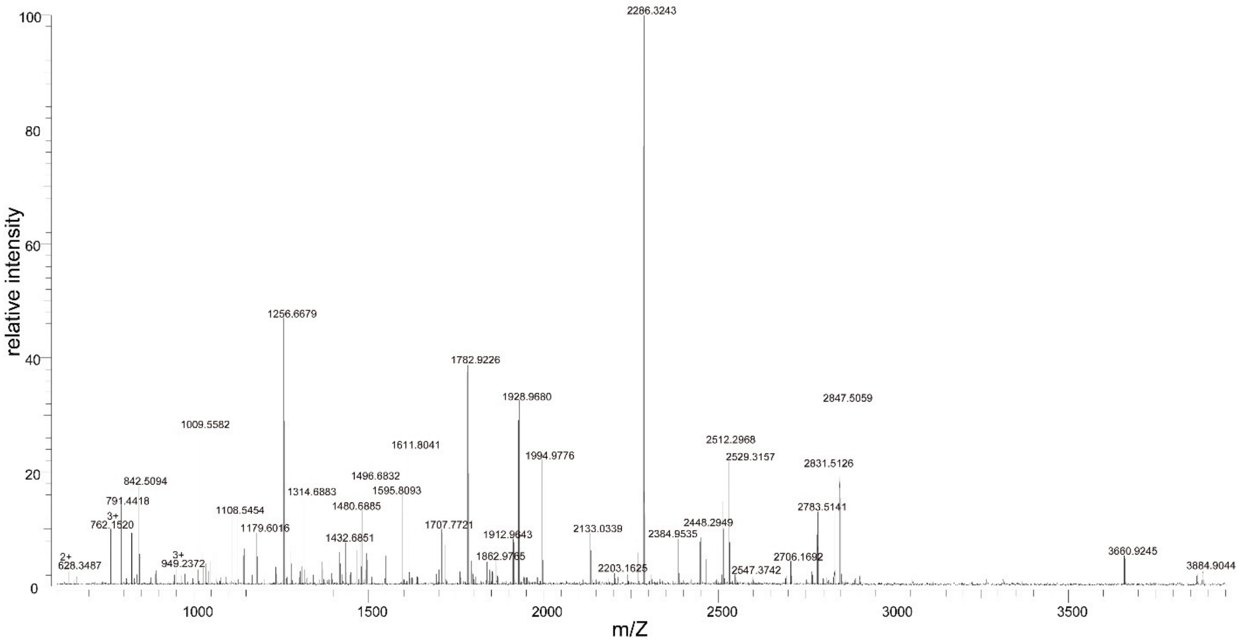

Spectrum of uncleaved *A. laidlawii* FtsZ.

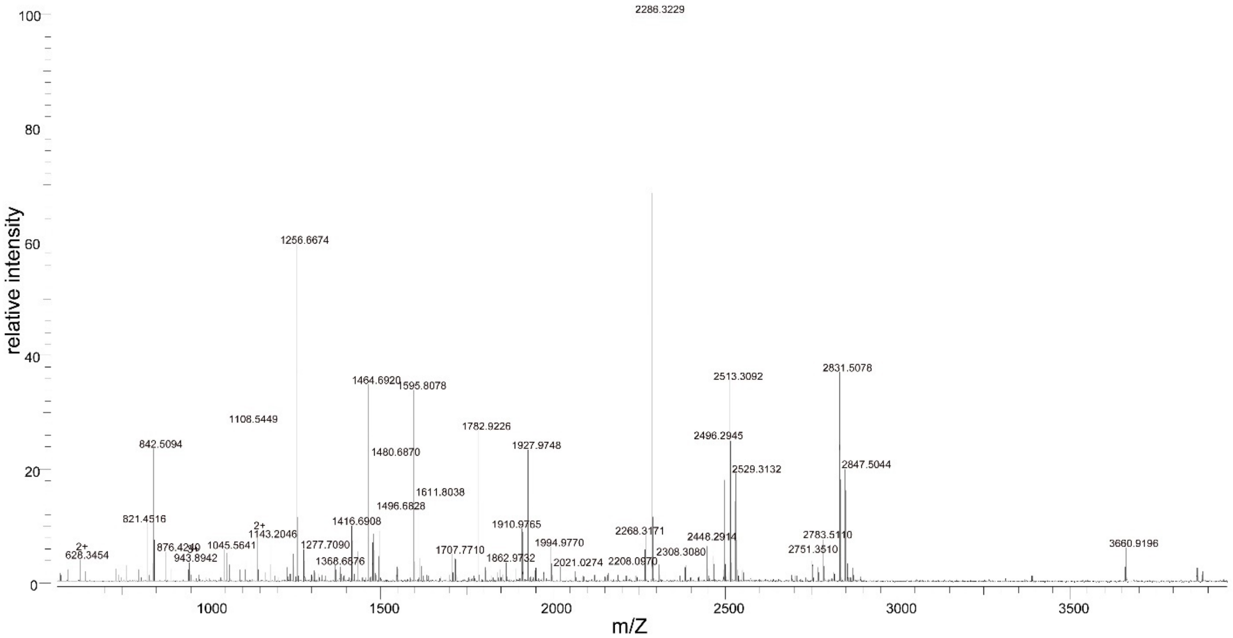

Spectrum of *A. laidlawii* FtsZ cleaved by a protealysin.
Full mass range.

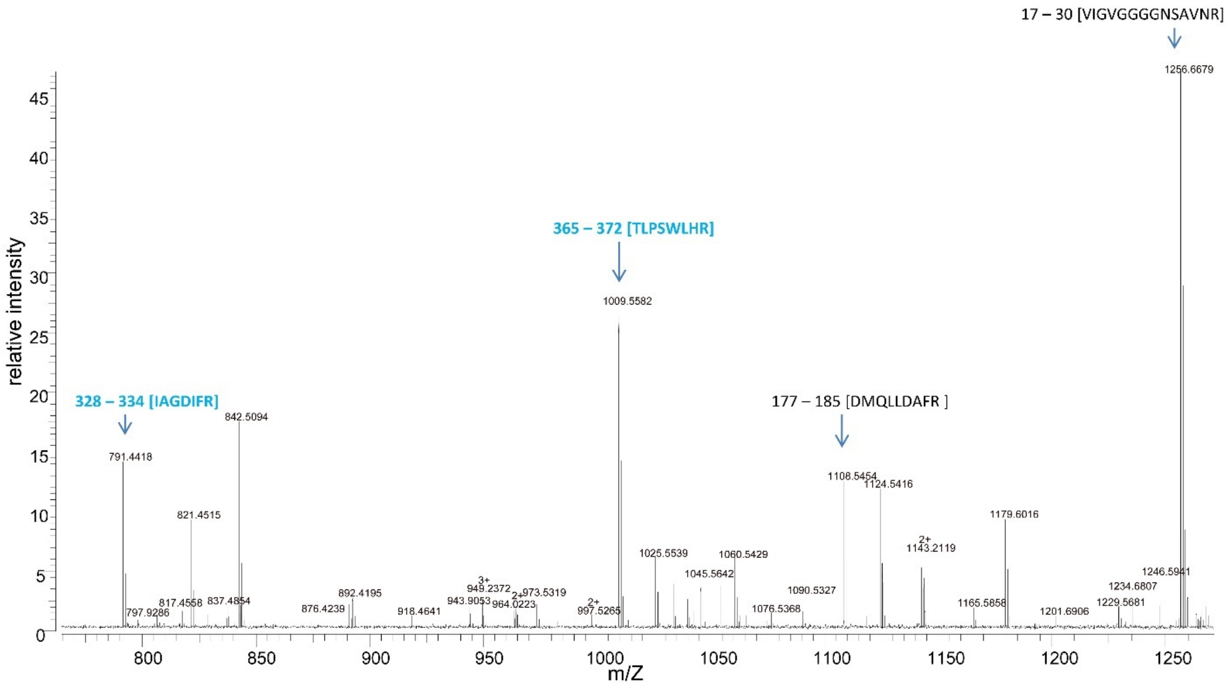

Spectrum of uncleaved *A. laidlawii* FtsZ.

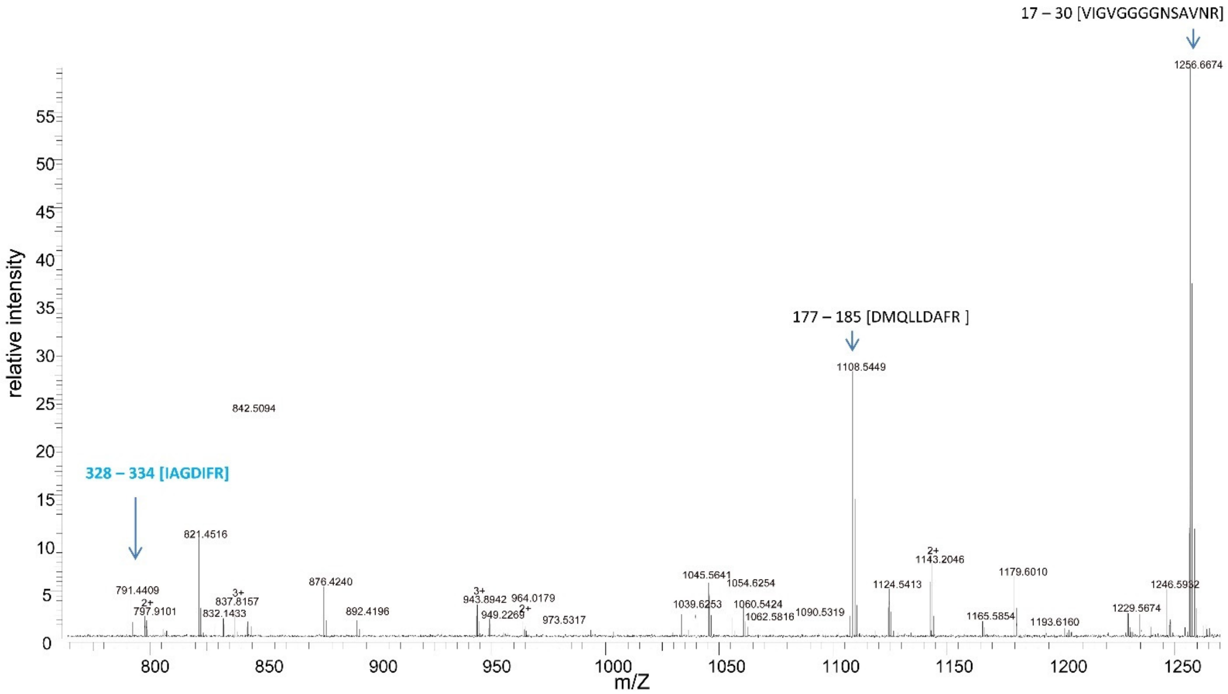

Spectrum of *A. laidlawii* FtsZ cleaved by a protealysin.

The spectra are shown with the selected mass range, in which the peaks of the cleaved protein disappear from the spectrum or the intensity of which is greatly reduced. Their corresponding peptides are marked in blue.

Supplementary S3. Peak intensities determined by mass spectrometry.

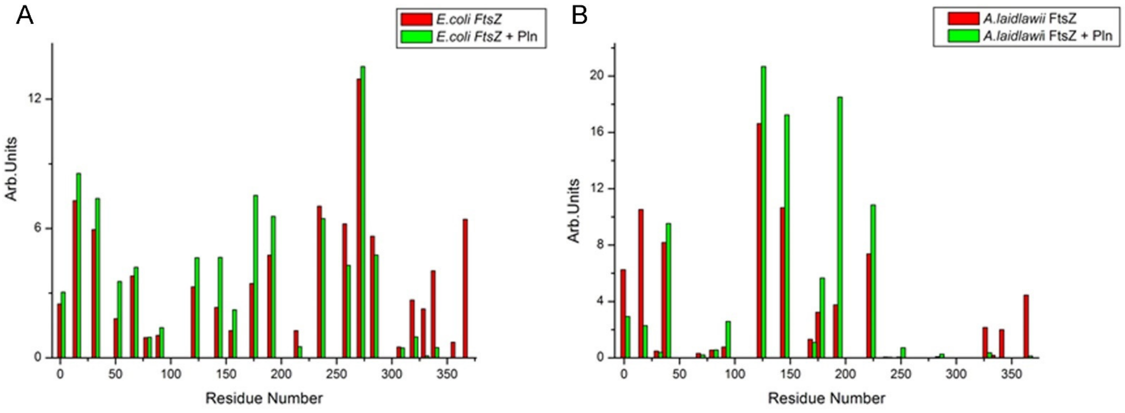

The abscissa shows the first amino acids numbers of the found peptides. The y-axis shows the average peak intensities corresponding to the found peptides in the spectra of the uncleaved FtsZ (red) and in the spectra of the FtsZ cleaved by a protealysin (green).

Supplementary S4. Peptides detected by mass spectrometry.

1	MFEPMELTND	AVIKVIGVGG	GGGNAVEHMV	RERIEGVEFF	AVNTDAQALR
51	KTAVGQTIQI	GSGITKGLGA	GANPEVGRNA	ADEDRDALRA	ALEGADMVFI
101	AAGMGGGTGT	GAAPVVAEVA	KDLGILTVAV	VTKPFNFEGK	KRMAFAEQGI
151	TELSKHVDSL	ITIPNDKLLK	VLGRGISLLD	AFGAANDVLK	GAVQGIAELI
201	TRPGLMNVDF	ADVRTVMSEM	GYAMMGSGVA	SGEDRAEEAA	EMAISSPLLE
251	DIDLSGARGV	LVNITAGFDL	RLDEFETVGN	TIRAFASDNA	TVVIGTSLDP
301	DMNDELRVTV	VATGIGMDKR	PEITLVTNKQ	VQQPVMDRYQ	QHGMAPLTQE
351	QKPVAKVVND	NAPQTAKEPD	YLDIPAFLRK	QAD	

Uncleaved *E. coli* FtsZ.

1	MFEPMELTND	AVIKVIGVGG	GGGNAVEHMV	RERIEGVEFF	AVNTDAQALR
51	KTAVGQTIQI	GSGITKGLGA	GANPEVGRNA	ADEDRDALRA	ALEGADMVFI
10	AAGMGGGTGT	GAAPVVAEVA	KDLGILTVAV	VTKPFNFEGK	KRMAFAEQGI
151	TELSKHVDSL	ITIPNDKLLK	VLGRGISLLD	AFGAANDVLK	GAVQGIAELI
201	TRPGLMNVDF	ADVRTVMSEM	GYAMMGSGVA	SGEDRAEEAA	EMAISSPLLE
251	DIDLSGARGV	LVNITAGFDL	RLDEFETVGN	TIRAFASDNA	TVVIGTSLDP
301	DMNDELRVTV	VATGIGMDKR	PEITLVTNKQ	VQQPVMDRYQ	QHGMAPLTQE
351	QKPVAKVVND	NAPQTAKEPD	YLDIPAFLRK	QAD	

E. coli FtsZ cleaved by a protealysin.

1	MVFGFNDEFN	QKPVIKVIGV	GGGGNSAVNR	MIENDVRGVS	YVAMNTDAQV
51	LKVSKADERI	QLGKKLTRGL	GAGAKPAIGK	QAALESEDDI	REVLSDADMV
101	FITAGMGGGT	GTGAAPVVAR	IAKELGVLTI	GIVTKPFVFE	GPLRMQHAIT
151	GLEELKPNVD	TLIVIPNERL	FSIADRDMQL	LDAFRESDKV	LRQGVQGIAE
201	IIAVPGMINV	DFADVRTVME	NKGTALMGIG	MASGENRAIE	AARKAIHSKL
251	LEVSIDGATD	AIVNISSGAE	VTLFEIEAAL	TEIRNATESD	LNVIYGHTVS
301	VDLEDEMIVT	IVATGYELRA	KGNEVEKIAG	DIFRNNSTQQ	VKITDTGLEP
351	LNNKEASGED	TKKRTLPSWL	HRK		

Uncleaved *A. laidlawii* FtsZ.

1	MVFGFNDEFN	QKPVIKVIGV	GGGGNSAVNR	MIENDVRGVS	YVAMNTDAQV
51	LKVSKADERI	QLGKKLTRGL	GAGAKPAIGK	QAALESEDDI	REVLSDADMV
101	FITAGMGGGT	GTGAAPVVAR	IAKELGVLTI	GIVTKPFVFE	GPLRMQHAIT
151	GLEELKPNVD	TLIVIPNERL	FSIADRDMQL	LDAFRESDKV	LRQGVQGIAE
201	IIAVPGMINV	DFADVRTVME	NKGTALMGIG	MASGENRAIE	AARKAIHSKL
251	LEVSIDGATD	AIVNISSGAE	VTLFEIEAAL	TEIRNATESD	LNVIYGHTVS
301	VDLEDEMIVT	IVATGYELRA	KGNEVEKIAG	DIFRNNSTQQ	VKITDTGLEP
351	LNNKEASGED	TKKRTLPSWL	HRK		

A. laidlawii FtsZ cleaved by a protealysin.

Peptides found by mass spectrometry are highlighted in color. Underlining indicates the amino acids after which the protein is cleaved by trypsin. Blue indicate peptides whose peak intensity in the spectrum of the cleaved FtsZ is 10 or more times lower than the intensity of these peaks in the spectrum of the uncleaved protein (Supplementary S2, S3). Examples of amino acids before which protealysin can cleave protein are highlighted in yellow [19].
